# Star wars against leukemia: attacking the clones

**DOI:** 10.1038/s41375-024-02369-6

**Published:** 2024-09-02

**Authors:** Monika M. Toma, Tomasz Skorski

**Affiliations:** 1 Fels Cancer Institute for Personalized Medicine, Lewis Katz School of Medicine, Temple University, Philadelphia, PA 19140 USA; 2 Department of Cancer and Cellular Biology, Lewis Katz School of Medicine, Temple University, Philadelphia, PA USA; 3 Nuclear Dynamics and Cancer Program, Fox Chase Cancer Center, Philadelphia, PA USA

**Keywords:** Targeted therapies, Translational research

## Abstract

Leukemia, although most likely starts as a monoclonal genetic/epigenetic anomaly, is a polyclonal disease at manifestation. This polyclonal nature results from ongoing evolutionary changes in the genome/epigenome of leukemia cells to promote their survival and proliferation advantages. We discuss here how genetic and/or epigenetic aberrations alter intracellular microenvironment in individual leukemia clones and how extracellular microenvironment selects the best fitted clones. This dynamic polyclonal composition of leukemia makes designing an effective therapy a challenging task especially because individual leukemia clones often display substantial differences in response to treatment. Here, we discuss novel therapeutic approach employing single cell multiomics to identify and eradicate all individual clones in a patient.

Carcinogenesis is a phenomenon where Darwinian principles are applicable to elucidate mechanisms responsible for intratumoral heterogeneity [1]. Cancer evolution is a process in which tumor cells adapt to the environment to promote the survivability and expansion of the best fitted clones [2–5]. This process depends on intracellular and extracellular factors [6–13]. Among intracellular factors, high demand for energy (ATP) and building materials (nucleic acids, amino acids, lipids, sugars), deregulated signaling pathways, accelerated cell cycle and protection from apoptosis generate metabolic and replication stress resulting in accumulation of spontaneous DNA damage and mutagenesis. Extracellular factors include, among other, hypoxia, stress, interaction with the microenvironment, microbiota, infection, inflammation, and antitumor immune response. The extra- and intra- cellular environmental pressure generates genetic and epigenetic alterations leading to clonal selection. Also, therapeutic regimens are powerful factors forcing genetic and epigenetic aberrations and clonal selection [14–16]. Tumor cells must adapt to these dynamic extra- and intra- cellular challenges resulting in expansion of the best fitted clones [17–20].

## Hallmarks of clonal evolution

Single cell “multiomics” approach [21] supported by computational power [22] revolutionized our perception of tumors [3, 6, 17], including myeloid malignancies [2, 5], which are highly dynamic polyclonal diseases. The interplay between genetic alterations (occurring spontaneously because of ongoing genomic instability, under the microenvironmental pressure, and after the treatment), heritable epigenetic modifications (occurring under the microenvironmental pressure and after the treatment) and immune responses produces malignant clones designed to thrive under specific conditions [23, 24].

**Fig. 1:**
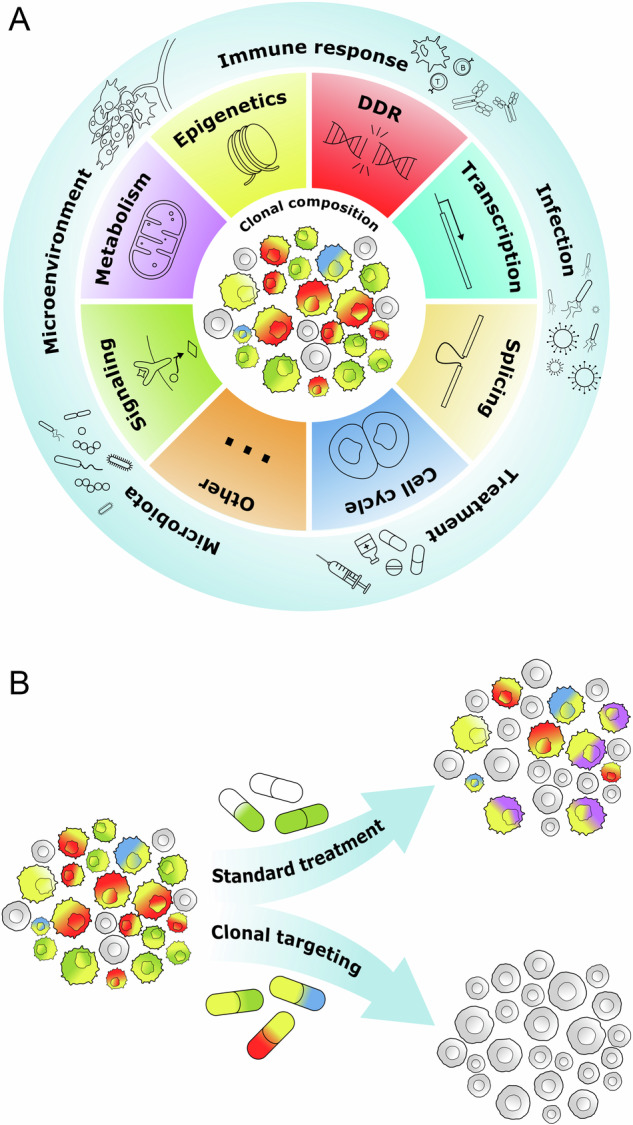
Hallmarks of cancer clonal heterogeneity and clonal attack strategy. **A** The interactions between extracellular factors (microenvironment, immune response/inflammation, infection, microbiota, and treatment) and intracellular factors (epigenetics, signaling, metabolism, DNA damage response = DDR, transcription, splicing, cell cycle, and other) generate unique clonal composition in individual tumors. **B** Standard treatment removes and/or reduces some clones, while others survive eventually facilitating the emergence of new clone(s). Conversely, clonal attack targets all clones at the same time thus eradicating the disease.

Genetic, epigenetic, proteomic, methylome and metabolomic aberrations define clonal functional heterogeneity and cooperativity which regulate clone-specific phenotypes such as selective microenvironmental advantages, resistance to immunological challenges and sensitivity to therapies [20, 24–26]. These ongoing fitness challenges are the hallmarks of malignant clonal expansion (Fig. 1A).

Clonal hematopoiesis with indeterminate potential (CHIP) is accompanied by emergence of clones carrying mutations in several genes such as *DNMT3A, TET2, JAK2, SF3B1, ASXL1, TP53, CBL, GNB1, BCOR, U2AF1, CREBBP, CUX1, SRSF2, MLL2, SETD2, SETDB1, GNAS, PPM1D*, and *BCORL1* and by increased rate of progression to hematologic malignancy [27]. CHIP is associated with aging, infections, chemotherapy and/or cigarette smoking, which alter tissue microenvironments to facilitate the selection and expansion of CHIP clones harboring specific mutations [28, 29]. For example, aging favors mutations in *SF3B1*, *SRSF2*, *ATM*, and *TET2* among others [28]. Inflammatory stress promotes expansion and further malignant transformation of hematopoietic clones carrying *TET2*, *DNMT3A*, and *ASXL1* mutations regulating DNA methylation and chromatin remodeling [29], and certain microbiota favor clones harboring *DNMT3A*, *FLT3*, and *NPM1* mutations [30]. Smoking promotes clones with *ASXL1*, *SRSF2*, *SF3B1*, and *JAK2* mutations [28]. On the other hand, expansion of the clones with *TP53* and *PPM1D* mutations which regulate DNA damage response is triggered by genotoxic stress and PARP inhibitor [14, 31, 32].

Remarkably, specific clonal advantages are often encoded by two, or more co-existing mutations. For example, *Flt3(ITD)* mutation generating an oncogenic tyrosine kinase (OTK) when combined mutations in *Dnmt3a* or *Tet2* in murine hematopoietic cells alter gene expression to an extend not seen with either mutation alone [20, 33, 34]. In addition, *FLT3(ITD);NRASmut* clone was selected under the treatment with FLT3 kinase inhibitor gilteritinib, whereas these mutations are usually mutually exclusive without the treatment [35]. Moreover, *KRAS* and *NRAS* mutations emerged in *JAK2* and *CALR* mutant clones during the treatment of myelofibrosis with JAK1/2 kinase inhibitor ruxolitinib [36].

It appears that the order of mutations acquisition is not random and some mutations in epigenetic modifiers like *DNMT3A*, *ASXL1*, *TET2* or *IDH1/2* are more likely to happen at the early stages, but *FLT3(ITD)* and mutations in *RAS* genes affecting intracellular signaling are more likely to happen at the late stages of transformation and disease progression [2, 37]. In addition, comprehensive study on 1540 AML patient samples showed a distinct, reoccurring co-mutation patterns. For instance, *NRAS(G12/G13)* but not *NRAS(Q61)* co-occurs with *NPM1* mutations. This confirms that the effect of mutations (even within the same gene) and their combinations might not have comparable effect [37]. Leukemogenesis is therefore an ordered and specific evolutionary process rather than an accidental chain of events.

Tumor cells and their surrounding microenvironment form a dynamic ecosystem [8]. Hypoxia, a hallmark of most tumors including leukemia in bone marrow niche, is a key factor in tumor microenvironment and can promote the outgrowth of tumor clones evading immune response and modulating their response to antitumor drugs [38, 39].

## Genetic and epigenetic aberrations regulate the response to treatment

There are numerous reports about how genetic and epigenetic aberrations regulate the sensitivity to a variety of drugs; the examples are summarized in Table 1. These discoveries led to successful clinical trials resulting in paradigm shifting on anti-tumor therapies.

**Table 1:** Intracellular and extracellular factors responsible for clonal composition of cancer.





*EA* epigenetic amplification, *ES* epigenetic silencing, *i* inhibitor, *MC* mutation change of function, *MG* mutation gain of function, *ML* mutation loss of function, *TKi* tyrosine kinase inhibitor.

For example, OTKs generated by abnormal activation due to mutations, translocations or amplifications are implicated in the induction, maintenance, malignant progression of cancer [40]. Therefore, OTKs have emerged as major targets for drug discovery which generated numerous FDA approved therapeutic modalities [41]. Another example of specific oncogene associated with particular treatment are acute promyelocytic leukemia cells expressing PML::RARA fusion protein which are very sensitive to *all-trans* retinoic acid and arsenic trioxide [42].

Exploring synthetic lethality as a therapeutic approach paved the way for new discoveries focused on cancer-related genomic and epigenomic abnormalities associated with the sensitivity to specific drugs [43]. In this field, synthetic lethality triggered by PARP inhibitors in *BRCA1/BRCA2*-mutated tumors revolutionized the treatment of breast and ovarian carcinomas, and also of other cancers carrying mutations in genes, which products are involved in homologous recombination (HR) [44]. However, mutations in *BRCA1/BRCA2* genes were detected only in 1-2% adult acute myeloid leukemia (AML) [45]. Moreover, genomic alterations in other DNA double strand breaks (DSBs) repair genes such as *ATM*, *PRKDC*, *ATR*, *RAD51*, and *RAD54* were reported in <1% acute leukemias.

Despite that mutations in DSBs repair genes are rare in hematological malignancies, PARP inhibitors found the application in triggering synthetic lethality in leukemias [46]. For example, gene expression and mutation analysis (GEMA) identified a cohort of acute leukemias displaying downregulated BRCA1/BRCA2 expression levels which were sensitive to PARP inhibitor [47]. In addition, *RUNX1::RUNX1T1, PML::RARA, IGH::MYC, TC3::HLF* translocations and *KDM6A*, *IDH1/2*, *TET2*, and *WT1* mutations compromised BRCA1/BRCA2-dependent HR-mediated repair of DSBs thus making leukemia cells sensitive to PARP inhibitor [34, 48–54]. These results provided foundation for numerous completed and ongoing clinical trials for PARP inhibitors in hematological malignancies [45, 46].

Moreover, BRCA1/BRCA2-deficiency and sensitivity to PARP inhibitor could be induced by FLT3 and JAK1/2 kinase inhibitors in AML and myeloproliferative neoplasm (MPN) cells expressing FLT3(ITD) and JAK2(V617F) OTKs, respectively [55]. The latter observation led to recent new clinical trial combining JAK2 inhibitor pacritinib and PARP inhibitor talazoparib in ruxolitinib-refractory MPN (NCT06218628).

In addition to DSB repair defects, dysregulation of other DNA damage response (DDR) mechanisms with potential therapeutic applications have been detected in leukemias. For example, mismatch repair (MMR) deficiencies due to somatic deletions of the genes that regulate MSH2 degradation were found in a cohort of newly diagnosed acute lymphoblastic leukemia (ALL) [56]. DNA polymerase beta inhibitor oleanolic acid treatment resulted in synthetic lethality in ALL with MMR deficiency through increased cellular apurinic/apyrimidinic sites, DNA strand breaks and apoptosis [57]. Also, AML cells displaying downregulation of the base excision repair (BER) glycosylase OGG1 were more sensitive to cytarabine [58]. Moreover, the odds of achieving complete remission in secondary AML patients were higher for the *XPD(D312N)* and *XPD(K751Q)* genotypes, previously associated with suboptimal nucleotide excision repair (NER) of more complex DNA lesions such as bulky DNA adducts [59, 60].

Epigenetics is dynamic and heritable modifications in DNA and histones that regulate gene activity independently of DNA sequence and can promote carcinogenesis and modulate drug response [61–63]. For example, loss of MLH1 and MGMT expression due to methylation resulted in resistance to platinum cytotoxic drugs and temozolomide, respectively [64, 65]. On the other hand, elevated expression of KDM5A caused resistance to cisplatin [66]. Thus, inhibitors of DNA methyltransferases (DNMTs), histone acetyltransferases and deacetylases (HATs and HDACs, respectively), and histone methyltransferases and demethylases (HMT and HDMT) have been developed and are used to treat cancer [67].

Potential clinical application of drugs targeting epigenetic modification such as DNMT and HDAC inhibitors has been explored in leukemias with limited success [68, 69]. The combinations of DNMT and/or HDAC inhibitors with other drugs, e.g., PARP inhibitors are under development [70, 71], but still lacking clear guidance about patient selection depended on genetic and epigenetic aberrations in leukemia. Recent studies with dual EZH1/EZH2 inhibitor valemelostat in adult T cell leukemia/lymphoma shed some light on mutation-dependent response [72]. For example, malignant clones with poor prognostic variations, including *PRKCB, TP53, IRF4* and *PDL1* responded well to the inhibitor, whereas these carrying *FN1, CCR4*, *MYO7B*, and *TET2* mutations were less sensitive/resistant.

The complexity of anti-tumor drug responses is multiplied by co-existence of the genetic and epigenetic aberrations which might generate mutational cooperativity linked to specific gain of functions [20, 73]. In addition, polyclonal composition of the majority of leukemias adds another challenge because individual clones may inherit a unique and overlapping genetic and epigenetic aberrations which regulate drug response in individual clones [74, 75]. Therefore, it is paramount the develop clonal biomarkers of the response to treatment.

## Clonal biomarkers defining the response to drugs

Genomic profiling identified pre-existing trackable sensitive and resistant tumor clones and revealed aberrations involved in drug response [76]. For example, clone carrying amplified *MET* was resistant to EGFR kinase inhibitor gefitinib and a clone with *CDKN2A* loss appeared sensitive to the combination of trametinib (MEK1/MEK2 kinase inhibitor) and palbociclib (CDK4/6 kinase inhibitor).

Regarding leukemia, AML clones with *IDH1* mutations seemed more sensitive to ABL1 kinase inhibitor ponatinib; conversely these with *NRAS* mutations were resistant to EGFR kinase inhibitor pelitinib [77]. Moreover, AML clones with *DNMT3A* mutations were resistant to anthracyclines, such as daunorubicin which combined with cytarabine is a part of 7 + 3 regimen [78]. In addition, AML cells carrying *RUNX1::RUNX1T1* and *CBFB::MYH11* translocations seemed more sensitive to the cytarabine [58]. Mutations in *SF3B1* and *TP53* were a risk factor for rapid clonal evolution and disease progression in chronic lymphocytic leukemia (CLL) patients treated with chemotherapy [79].

Malignant clones usually carry more than one mutation which may interfere with each other. For example, while AML clones expressing FLT3(ITD) kinase seemed more responsive to the kinase inhibitor crizotinib, co-occurrence of *DNMT3A* mutation predicted resistance not only to the kinase inhibitor, but also to axitinib (P-glycoprotein efflux transporter inhibitor), cediranib (VEGFR inhibitor), ponatinib (ABL1 kinase inhibitor) and tofacitinib (JAK1/3 kinase inhibitor) [77].

AML and MPN cells usually accumulate spontaneous DNA damage, including highly toxic DSBs, induced by metabolic products and replication stress [55, 80, 81]. AML and MPN clones activate DDR mechanisms which are regulated by clone-specific mutations to survive endogenous genotoxic stress suggesting that DDR is a legitimate therapeutic target. For example, *FLT3(ITD), JAK2(V617F), MPL(W515L), CALR(del52), TETmut, DNMT3Amut, RUNX1::RUNX1T1, RML::RARA, SRSF2mut, IDH1mut*, and *KITmut* regulate DDR and affect the response of leukemia cells to PARP, RAD52 and Polθ inhibitors by modulating the expression of DNA repair proteins such as BRCA1 and BRCA2 (involved in HR) and Polθ (a key factor in microhomology-mediated end-joining) [34, 47, 49, 81–83].

Again, these mutations might interfere with each other to modulate DSB repair [34, 55, 81, 84, 85]. While *TET2mut* clones carrying *FLT3(ITD), NRAS, JAK2(V617), CALRmut or MPLmut* were sensitive to PARP inhibitors, the counterparts carrying *DNMT3Amut* were resistant [34]. Remarkably, *FLT3(ITD);DNMT3Amut;TET2mut* clone regained the sensitivity to PARP inhibitors. Moreover, *RUNX1-RUNX1T1;FLT3(ITD)* clones were sensitive to PARP inhibitor, conversely *RUNX1::RUNX1T1; KITmut* were resistant [84, 85]. *CBFB::MYH11; KITmut* and *CBFB::MYH11; NRASmut* were also associated with clonal resistance and sensitivity, respectively, to PARP inhibitor but not to doxorubicin [85].

Altogether, these results highlight the complexity of genetic signatures regulating clonal response to genotoxic agents, kinase inhibitors and DDR inhibitors. Single cell “omics” (e.g., transcriptomics, genomics, epigenomics, proteomics, metabolomics) and more recently single cell “multiomics” simultaneously integrating various “singleomics” should help to better understanding of cancer polyclonal composition and therapeutic vulnerabilities [26, 86]. Recently, combined analyses of numerous scRNAseq databases precipitated in the generation of artificial intelligence (AI) -powered PERCEPTION approach to predict responses to targeted therapies if multiple myeloma and breast cancer [87].

## Clonal attack

Personalized medicine is an approach to tailoring disease prevention and treatment that considers differences in patient’s tumor genetic/epigenetic/metabolomic/immunologic characteristics. Clonal medicine is an innovative approach to tailoring the treatment that eliminates tumor clones based on clone’s unique genetic/epigenetic/metabolomic/immunologic makeup.

Tumor evolution is responsible for cancer heterogeneity which has to be accounted for when planning a therapeutic approach [88, 89]. Variabilities in tumor clonal response to the treatment has been suggested more than 45 years ago [90]. Tumor clones respond differently to various drugs due to their unique and overlapping “multiomics” properties. Thus, a successful therapeutic regimen must simultaneously eradicate all malignant clones (Fig. 1B).

For example, AML and MPN are heterogenous diseases, and clonal heterogeneity hampers the effectiveness of therapeutics against these hematological malignancies [2, 5]. Clones within a leukemia sample demonstrate differences in response to current drugs leading to disease relapse from the pre-existing clones selected by the treatment and/or those induced under the therapeutic pressure due to continuous clonal evolution [5, 91, 92]. The genetic landscape of malignant clones in a patient may be complicated since individual clones can carry multiple mutations, both overlapping and unique for the individual clones [93].

Recently, we designed a successful multiclonal attack to eradicate AML and MPN clones which accumulate DNA damage due to continuous metabolic stress [94]. We showed that a combination of DDR inhibitors simultaneously attacking all clones in a patient sample eradicated the disease in vitro and in vivo. The “clonal attack” by DDR inhibitors shifts the paradigm of genotoxic therapies from those using non discriminative cytotoxic drugs to those selectively attacking DDR vulnerabilities in AML and MPN clones. Therapeutic potential of the combinatorial effects of FDA approved drugs (e.g., tyrosine kinase inhibitors, and hypomethylating, genotoxic and pro-apoptotic agents) with DDR inhibitors need to be explored to evaluate if standard treatment enhance the effectiveness of DDR inhibitors in combating malignant clones.

Since clonal heterogeneity and DNA damage are hallmarks of cancer, the “clonal attack” may open new era in cancer treatment and be broadly applicable to the quest for cure [3, 17]. However, integration of the “clonal attack” into everyday clinical practice would require two steps. First, an extensive experimental single cell “multiomics” data must be gathered to generate enough information for artificial intelligence (AI) to effectively resolve tumor clonal composition and predict individual clones’ sensitivity to therapeutic interventions [95]. Second, hospitals must have the personnel and equipment to be able to perform “multiomic” analysis and interpret the data on daily basis.
